# Gemcitabine and oxaliplatin (GEMOX) in gemcitabine refractory advanced pancreatic adenocarcinoma: a phase II study

**DOI:** 10.1038/sj.bjc.6602966

**Published:** 2006-01-24

**Authors:** A Demols, M Peeters, M Polus, R Marechal, F Gay, E Monsaert, A Hendlisz, J L Van Laethem

**Affiliations:** 1 Department of Gastroenterology, GI Oncology Unit, Erasme University Hospital, Route de Lennik 808, Brussels 1070, Belgium; 2 Department of Gastroenterology, UZ Gent, 9000 Gent, Belgium; 3 Department of Gastroenterology, CHU Sart Tilman, 4000 Liège, Belgium; 4 Department of Gastroenterology, Institut Bordet, 1000 Brussels, Belgium

**Keywords:** advanced and metastatic pancreatic cancer, gemcitabine plus oxaliplatin, second line chemotherapy

## Abstract

Gemcitabine and oxaliplatin (GEMOX) are active as first-line therapy against advanced pancreatic cancer. This study aims to evaluate the activity and tolerability of this combination in patients refractory to standard gemcitabine (GEM). A total of 33 patients (median age of 57) were included with locally advanced and metastatic evaluable diseases, who had progressed during or following GEM therapy. The GEMOX regimen consisted of 1000 mg m^−2^ of GEM at a 100-min infusion on day 1, followed on day 2 by 100 mg m^−2^ of oxaliplatin at a 2-h infusion; a cycle that was given every 2 weeks. All patients received at least one cycle of GEMOX (median 5; range 1–29). Response by 31 evaluable patients was as follows: PR: 7/31(22.6%), s.d. ⩾8 weeks: 11/31(35.5%), s.d. <8 weeks: 1/31(3.2%), PD: 12/31(38.7%). Median duration of response and TTP were 4.5 and 4.2 months, respectively. Median survival was 6 months (range 0.5–21). Clinical benefit response was observed in 17/31 patients (54.8%). Grade III/IV non-neurologic toxicities occurred in 12/33 patients (36.3%), and grade I, II, and III neuropathy in 17(51%), 3(9%), and 4(12%) patients, respectively. GEMOX is a well-tolerated, active regimen that may provide a benefit to patients with advanced pancreatic cancer after progression following standard gemcitabine treatment.

Locally advanced and metastatic (MET) pancreatic adenocarcinomas carry a very poor prognosis. In patients treated with the standard palliative treatment gemcitabine (GEM), median survival still remains only 6 months (Burris *et al*, 1997). Over the last several years, many trials have been designed combining GEM with various other drugs to treat chemo-naive patients, with the aim to improve overall survival (OS) (Table 1). Unfortunately, none of the GEM-based combinations studied so far have reached that objective, with the exception of GEM plus Erlotinib, which showed a slight increase in OS to 6.4 months (Moore *et al*, 2005). However, some trials – mainly those using platinum based combinations – have shown an increase in response rate (RR) and time to progression (TTP) (Table 1).

Unsurprisingly, the combination of GEM administered at a fixed dose rate (FDR) together with oxaliplatin (OX) is being tried more and more. It has become apparent that a FDR of 10 mg m^−2^ min^−1^ GEM is the optimal infusion speed to achieve the best conversion rate of active phosphorylated gemcitabine, providing a two-fold increase in intracellular gemcitabine triphosphate concentration (Tempero *et al*, 2003). Tempero *et al* have shown that when doing this, median survival was 8.0 months in the FDR arm (*P*=0.013), and only 5.0 months in the standard arm (30-min infusion). In addition, at this infusion rate, GEM can be safely combined with oxaliplatin, with no overlapping toxicity. This combination appears to produce a sequence-dependent synergy of activity when exposing tumour cells firstly to GEM and then 24 h later with oxaliplatin (Faivre *et al*, 1999).

The combination of GEM and oxaliplatin (GEMOX regimen) has been reported by Louvet *et al*, to be active in first-line therapy against advanced and metastatic pancreatic cancer. GEMOX has also been shown to provide significantly better RR, clinical benefit response (CBR) and period-free survival (PFS) than GEM alone (Louvet *et al*, 2005). Furthermore, toxicity with this combination is limited (Louvet *et al*, 2002).

The GEMOX combination was shown to have a survival benefit of an additional 2 months, but this difference was not significant. Possible causes of this could be the lack of power in the statistical assumption, the inclusion of a high number of locally advanced diseases (LAD), and the proportion of second-line therapy using a platinum-based regimen.

However, in light of these interesting results, there are still no guidelines or recommendations for selecting treatment for patients progressing after GEM therapy; nor are there any reports of regimens with demonstrated activity that would enable one to justify one approach over another.

Consequently, we designed a multicentre phase II feasibility study whose primary aim was to evaluate activity and tolerability of the GEMOX regimen as second-line chemotherapy in gemcitabine-refractory advanced and metastatic pancreatic cancer. Secondary aims of the study included evaluation of TTP and overall survival.

## PATIENTS AND METHODS

### Patient eligibility

The study has been approved by the ethics committees of each participating centre.

A total of 33 patients were eligible in four Belgian academic hospitals. Inclusion criteria were: histologically proven locally advanced or metastatic pancreatic adenocarcinoma, and progressive disease during or within 3 months of gemcitabine as first-line therapy or in adjuvant setting (including combination with radiation). The disease had to be measurable according to modified RECIST criteria (Therasse *et al*, 2000). All patients signed a written informed consent to participate, were over 18 years old, had a World Health Organization (WHO) performance status (PS) of 0, 1 or 2, and a life expectancy of more than six weeks (due to bad prognosis of the disease). Adequate bone marrow (ANC⩾1.5 × 10^9^ L^−1^, platelets ⩾100 × 10^9^ L^−1^, Hb ⩾9 g dl^−1^), liver function (AST, ALT ⩽2 × ULN, total bilirubin ⩽1.5 × ULN), and renal function (serum creatinin ⩽2 mg dl^−1^) were required.

Previous or concomitant malignancy, cystic or neuroendocrine tumours, and peripheral neuropathy (regardless of origin) were considered as exclusion criteria.

At the time of inclusion, all patients underwent clinical examination. Weight, height and PS were carefully recorded, and abdominal CT, chest X-ray and plasma CA19.9 measurement were performed before GEMOX administration.

### Treatment plan

GEMOX regimen (Louvet *et al*, 2002) consisted of 1000 mg m^−2^ of GEM at a 100-min infusion on day 1 (infusion rate 10 mg m^−2^ min^−1^), followed on day 2 by 100 mg m^−2^ of oxaliplatin at a 2-h infusion. This cycle was given every 2 weeks until progression of the disease. Oxaliplatin was provided by Sanofi-Synthelabo (Paris, France) on a compassionate use basis.

### Dose modifications

Before each cycle, assessment of haematologic and nonhaematologic toxicities were performed using the NCI-CTC toxicity scale (version 2.0).

In cases of febrile neutropenia or bleeding due to thrombocytopenia, treatment was permanently withheld.

In cases of non-neurologic toxicity above grade (gr) 2, the entire next cycle was delayed until toxicity had declined to gr 2 or less. After recovery, subsequent doses were reduced as follows: GEM 800 mg m^−2^ at an 80-min infusion and oxaliplatin 85 mg m^−2^ at a 2-h infusion.

If a gr 3 cumulative typical peripheral neuropathy appeared, oxaliplatin was discontinued; in the case of gr 2, the dose was reduced to 85 mg m^−2^. For laryngopharyngeal dysesthesia, oxaliplatin infusion was reduced and given 6 h – and eventually stopped if further symptoms occurred during the following cycles.

### Concomitant therapies

During the entire treatment, patients could receive full supportive care: antiemetics (anti-5HT_3_), steroids, analgesics, antibiotics, and acid-secretion inhibitors were permitted at the discretion of the physician in charge of the patient. No other chemo-, immuno- or experimental therapy was allowed during the study.

### Follow-up

Until progression of the disease, Karnovsky Assessment (KA), physical examination, and blood analysis (haematology and chemistry tests) were performed before the administration of each cycle.

NCI-CTC toxicity scale (version 2.0) haematologic, non-haematologic, and neurologic (Sanofi scale) toxicities were carefully recorded, as well as late toxicities, if any.

Clinical benefit was evaluated weekly, and was based on KS assessment, weight measurement, evaluation of pain intensity (using a visual analogue scale), and analgesic consumption. Patients were asked to fill out a daily diary, which was reviewed during each patient's weekly assessment.

Evaluation of the tumour response was performed every four cycles (or at anytime if there was clinical evidence of progression) using modified RECIST criteria. Treatment was stopped at progression of the disease.

Patients were followed until death.

### Statistical analysis

The number of the patients required for the study has been determined according to a two-stage Simon design. The target enrolment was estimated to be 33 patients, and all analyses were performed on the intention-to-treat population.

The minimum target activity level was 10% and early discontinuation of the trial was planned in the case of only two or fewer patients responding to treatment in the first 27 patients assessed. This reduces the chance of inadvertently rejecting a true response, or including a response rate below a certain standard. The trial design, therefore, ensures that (i) there is no more than a 10% chance that a treatment with a true response rate (25% or more) is rejected, and (ii) that there is no more than a 10% probability that a response rate of 10% or less would be accepted.

The primary end point of the study was activity measured by RR and the feasibility of this second-line combination, where feasibility was defined as toxicity and tolerance.

In addition, as secondary end points, we evaluated the clinical benefit response, time to progression (TTP), and survival. Survival was calculated from the start of GEMOX administration until the death of the patient.

Statistical analysis was performed using the SPSS program (SPSS, Chicago, IL, USA).

## RESULTS

### Patient's population

A total of 33 patients (17 men and 16 women) with a median age of 57 (range 27–76), and WHO PS scores of 0/1/2 (12, 17, and four patients, respectively) were included in a 12-month period. There were 12(35%) with locally advanced disease and 21(65%) with metastatic measurable disease. All 33 patients had previously progressed during or following GEM-based therapy (GEM alone: 14 patients, GEM+other drug: 10 patients, and GEM+radiation (RT): nine patients – including six adjuvant treatments after surgery, where all patients showing recurrence after adjuvant therapy (ADJ) discontinuation did so within 3 months). No patient received gemcitabine as a fixed dose rate infusion during the first-line treatment. Detailed patient demographics and prior therapy are listed in Table 2.

### Treatment completion and dose reductions

All patients received at least one cycle of GEMOX. A total of 208 cycles were administrated, with a median number of five cycles given (range 1–29 cycles).

Eleven cycles were delayed due to gr 3 or 4 toxicities (described in Table 3). After recovery from toxicity, subsequent cycles were administered at reduced doses in 11 patients, and no additional delay or dose reduction was necessary. Of the 208 cycles given, 29(13.9%) were administered at reduced doses.

Definitive withdrawn of treatment due to gr 3 peripheral neuropathy occurred in four patients (12%) after 8, 8, 9, and 10 cycles of GEMOX.

### Toxicity

One toxic death was reported. This was due to febrile neutropenia that occurred after five cycles of GEMOX (without any previous haematologic toxicity). This patient did have a response to GEMOX after four cycles of treatment, but this was, of course, not confirmed and not considered as a PR.

Haematologic and nonhaematologic toxicities are described in Table 3.

One gr 4 toxicity was reported, consisting of febrile neutropenia that lead to the death of the patient. Gr 3 haematologic toxicities were observed in 8/33 patients (24%), consisting of neutropenia (3), anaemia (2), and thrombopenia (3). Gr 3 nonhaematologic toxicity occurred only in three patients (11%): nausea (two patients) and vomiting (one patient).

Grade 1, 2, and 3 peripheral neuropathy were observed in 17(51%), 3(9%), and 4(12%) of 33 patients, respectively.

The total number of gr 3–4 toxicities recorded was 16 patients (48%).

Overall, the regimen was well tolerated.

### Efficacy results

Of the 33 patients, 31 were evaluable for response and clinical benefit response.

Seven patients (22.6%) experienced a partial response (PR) that was confirmed, with a median duration of this response of 4.5 months (range 1.5–16).

In all, 12 patients (38.7%) were stabilised (stable disease, s.d.), whereby s.d. endured for more than 8 weeks in 11 patients (35.5%), and for <8 weeks in 1/31 (3.2%).

Median TTP was 4.2 months. Median survival since the start of GEMOX was 6 months (range 0.5–21). Median survival since the start of first-line treatment was 12 months (range 3–34).

A clinical benefit response was observed in 17 evaluable patients (54.8%).

Regarding the 16 patients who had progressed within 3 months of previous first-line GEM, best responses were: 1 PR, 5 s.d. and 10 PD. Re-challenging this group of patients with GEMOX led to PR in 1/1, 3/5, and 3/10, respectively, and s.d. in four additional patients.

## DISCUSSION

The present study reports, for the first time, the use of the GEMOX regimen in gemcitabine-refractory, locally advanced and metastatic pancreatic adenocarcinoma. Currently, in gemcitabine-refractory patient, there is no data concerning the choice of the second-line therapy. For these patients, we chose to assess the GEMOX regimen based on its activity (28.7%) and tolerability profile reported in first-line use (Louvet *et al*, 2005). The schedule of administration and infusion rate within our study were similar to previous preclinical and clinical data (Faivre *et al*, 1999; Tempero *et al*, 2003). We chose to include LAD, because our trial design was a feasibility phase II study, and not a phase III survival trial.

The tolerability of GEMOX is well assessed as 59% of were considered to be in poor condition (PS 1–2), where no major toxicities were seen. Overall, treatment was well tolerated, and with the exception of one gr 3 neuropathy and one febrile neutropenia, no other toxic event led to treatment interruption. Acute haematologic toxicity was limited, consisting mainly in gr 3 anaemia, thrombopenia and neutropenia. One toxic death was reported due to a febrile neutropenia that occurred after five cycles of GEMOX without any previous haematologic toxicity during the four first administrations. Typical peripheral oxaliplatin-related neuropathy was noted: half of the patients experienced a gr 1 neurotoxicity and four patients (12%) had developed gr 3 neuropathy leading to withdrawal of GEMOX treatment. In these four particular patients, GEMOX was stopped after 8, 8, 9, and 10 cycles, respectively. Globally, these toxicity data are comparable to those from other studies using GEMOX in first line (Table 4).

The activity of the regimen was good, achieving partial response in 22.6% of and disease stabilisation for more than 8 weeks in 35.5% of patients. This activity is comparable to that observed in first line by Louvet (28.7%) and to that observed in second line using oxaliplatin (50 mg m^−2^ weekly)/LV/5-FU (Tsavaris *et al*, 2005). A 6-month survival can be expected in the setting of second-line therapy, as supported by the findings of this study and other phase II trials based with other drugs combinations (Table 5).

Manageable toxicity and good clinical benefit response rate were also important valuable findings of this trial.

Our data also suggest that the addition of oxaliplatin to gemcitabine may be active and effective, even after progression or failure under the latter drug. Currently, it is difficult to differentiate between the relative role of each drug in overcoming the tumour resistance to gemcitabine administered alone in a standard 30-min regimen. The addition of oxaliplatin to gemcitabine on day 2 could explain this resensitisation of the tumour, but the slower rate of infusion of gemcitabine versus how it was administered in first-line might also play a role in this recovered activity. Additional data is needed to determine what gives GEMOX its incremental activity over GEM in second-line.

Results coming from the ECOG 6201 trial, comparing gemcitabine as a standard 30-min infusion, gemcitabine at a fixed-dose rate, and the GEMOX regimen will determine the best way to administer gemcitabine. It is anticipated that this trial will also clarify the role of oxaliplatin when added to gemcitabine in chemo-naive patients. The Louvet trial has shown a better activity profile of the combination of these drugs versus monotherapy, but has failed to demonstrate a clear statistically significant survival advantage. These results are key to determine the place of oxaliplatin in the management of advanced pancreatic cancer.

Recently, other data have suggested a beneficial role of oxaliplatin in second-line therapy using the OFF regimen (oxaliplatin/folinic acid/5-FU 24 h). The randomised CONKO 003 phase III trial showed a statistically significant increase in overall survival when giving OFF regimen in second line, as compared to best supportive care (Oettle *et al*, 2005).

Nonetheless, many issues remain unanswered in the management of pancreatic cancer. The need to determine prognostic and predictive factors is essential to identify the best therapy for all patients. The emergence of biological therapies will, in fact, require such a prospective selection of patients based on these factors. The combination of new biological agents with another cytotoxic, such as oxaliplatin to gemcitabine could become a treatment standard. In the setting of second-line therapy, a prospective well-designed phase III trial would be useful to validate oxaliplatin-based regimens. In this setting, GEMOX could be compared to BSC, or to another oxaliplatin-based regimen; FOLFOX for example.

Our study, showing that the GEMOX regimen is active and well tolerated after progression with standard gemcitabine therapy, supports the beneficial role of this combination in advanced pancreatic cancer.

## Figures and Tables

**Table 1:** Recent randomised phase III studies using gemcitabine-based combinations in first-line therapy

**Regimen**	**Authors**	**OS (months)**	**RR (%)**	**TTP (months)**
GEM	Burris *et al* (1997)	5.6*	5.4*	2.5
5-FU		4.41	0	0.9
				
GEM	Louvet *et al* (2005)	7.1	16.7	3.7
GEMOX		9.0	28.7*	5.8
				
GEM	O'Reilly *et al* (2004)	6.2	6.3	3.8
GEM-DX		6.7	8.2	3.7
				
GEM	Rocha Lima *et al* (2004)	6.6	4.4	3.0
GEM-CPT11		6.3	16.1*	3.4
				
GEM	Heinemann *et al* (2003)	6.0		2.5
GEM-CDDP		8.3		4.6
				
GEM	Richards *et al* (2004)	6.3	9.1	3.6
GEM-pemetrexed		6.2	18.3*	5.2^*^
				
GEM	Moore *et al* (2005)	5.91	8.0	3.55
GEM-Erlotinib		6.4*	9.0	3.75

Recent randomised phase III studies using gemcitabine-based combinations in first-line therapy.

* *P*<0.05.

**Table 2:** Patients' demographics and prior therapy

**Characteristics**	***n***
Men/women	17/16
PS 0/1/2	12/17/4
LAD/MET	12/21
Adjuvant RT-CT	6
	
*Prior therapy*	
RT-CT	3
GEM	14
GEM-PX	7
GEM-CDDP	2
Ralitrexed-GEM	1

Patients' characteristics and first line-therapy received. MET: metastatic disease; LAD locally advanced disease.

**Table 3:** Reported haematologic and nonhaematologic toxicities

***n*=33**	**Neutropenia**	**Anaemia**	**Thrombopenia**	**Total**		
*Haematologic toxicity*						
Grade 1	1	3	6	10		
Grade 2	4	0	0	4		
Grade 3	3	2	3	8		
Grade 4	1	0	0	1		
All grades	9	5	9	23		
						

***n*=33**	**Nausea**	**Vomiting**	**Diarrhea**	**Fatigue**	**Neurotox**	**Total**
*Nonhaematologic and neurologic toxicity*						
Grade 1	5	4	0	7	17	33
Grade 2	3	3	1	4	3	14
Grade 3	2	1	0	0	4	7
Grade 4	0	0	0	0	—	0
All grades	10	8	1	11	24	54

Reported haematologic and nonhaematologic toxicities.

**Table 4:** Comparison of toxicity profiles (values are expressed in %)

**Toxicity (in %)**	**Demols (second line)**	**Louvet (2)**	**Heinemann (10)**
Gr3 neuropathy	12	19	ND
Gr3-4 neutropenia	12.5	14	5
Gr3-4 anaemia	6	8	0
Gr3-4 thrombopenia	9	17	15
Gr3-4 nausea and vomiting	9	11	ND

Comparison of toxicity profiles (values are expressed in %). ND: not determined.

**Table 5:** Phase II and III trials in second-line therapy

**Regimen**	**Authors and phase**	**Number of patients**	**PR (%)**	**s.d. (%)**	**Median OS (weeks)**
OX/LV/5-FU	Tsavaris	30	23.3	30	25
	Phase II				
					
OX	Androulakis *et al* (2005)	18	0	16.7	ND
	Phase II				
					
CPT-11/ralitrexed vs ralitrexed	Ulrich-Pur *et al* (2003)	38	16	ND	26
	Ramdomised II		0	ND	17
					
Paclitaxel	Oettle *et al* (2000)	18	5.5	27.7	17.5
	Phase II				
					
Capecitabine/erlotinib	Blaszkowsky *et al* (2005)	30	11	57	27
	Phase II				
					
OX/FA/5-FU	Oettle *et al* (2000)	46	ND	ND	21
Vs BSC	Phase III				10
					
CPT-11/FA/5-FU	Ng *et al* (2005)	15	0	38	14
	Phase II				
					
GEMOX	Demols				
	Phase II	33	22.6	38.7	25

Phase II and III trials in second-line therapy. BSC: best supportive care; ND: not determined.
